# Excellent Results in the Off-Label Use of Denosumab in Pediatric Oncology: Monocentric Case Series Study

**DOI:** 10.1155/crpe/6693304

**Published:** 2025-12-03

**Authors:** Teresa Battaglia, Marta Nebiolo, Massimo Conte, Virginia Livellara, Gianluca Piatelli, Maria Beatrice Damasio, Barbara Galleni, Elena Arkhangelskaya, Carla Manzitti, Gabriele Gaggero, Alberto Garaventa

**Affiliations:** ^1^Department of Hemato-Oncology, Oncology Unit, IRCCS Giannina Gaslini Institute, Genoa, Italy; ^2^Department of Neurosciences, Rehabilitation, Ophthalmology, Genetics, Maternal and Child Health, University of Genoa, Italy IRCCS Giannina Gaslini Institute, Genoa, Italy; ^3^Department of Surgical Sciences, Neurosurgery Unit, IRCCS Giannina Gaslini Institute, Genoa, Italy; ^4^Department of Services, Radiology Unit, IRCCS Giannina Gaslini Institute, Genoa, Italy; ^5^Department of Services, Pathology Unit, IRCCS Giannina Gaslini Institute, Genoa, Italy

## Abstract

Denosumab is a human monoclonal antibody approved by the Food and Drug Administration in 2013 for use in adults with inoperable giant cell tumor of bone (GCTB). In children, it is used as an off-label drug in some giant cell-rich tumors of bone (GCRTB) and giant cell granuloma (GCG). The aim of the study is to evaluate the efficacy and acute toxicity of denosumab in children. From February 2022 to December 2023, at the Istituto Giannina Gaslini, 5 patients were treated with denosumab, 2 females and 3 males. Age ranged from 8 to 17 years. Two had ABC (hemipelvis and D9), and 3 had GCG (sphenoid, jaw, and maxilla). An assay of calcium and vitamin D as well as a dental scan was performed before administering denosumab due to possible side effects such as hypocalcemia and jaw osteonecrosis. Therapy was given subcutaneously at the dose of 70 mg/m^2^ (weight < 50 kg) or 120 mg (weight > 50 kg) at days +1, +8, +15, +28, and then monthly for 3–5 months, followed by imaging evaluation. Overall, 21 doses were administered to Patient 1, 20 doses to Patient 2, 11 doses to Patient 3, 10 doses to Patient 4, and 9 doses to Patient 5. Calcium carbonate and vitamin D were given as supportive therapy. We observed lesion volume reduction in 4 patients and radiological stability in 1 patient. Surgery was possible in 1 case thanks to the significant reduction of lesion size. A longer administration over 22 months was safe and well tolerated. No disease progression or side effects were observed. This study confirms literature data about the use of denosumab in inoperable GCRTB. These results are preliminary; further studies are necessary on a larger series of cases, with a longer follow-up (3–5 years), with data collection even from other pediatric centers.

## 1. Introduction

Denosumab is an IgG2 monoclonal antibody that inhibits receptor activator of nuclear factor-kappa β ligand (RANKL), inhibiting osteoclastogenesis directly. Generally used to treat osteoporosis, denosumab was approved by the Food and Drug Administration in 2013 and by the European Medicines Agency in 2014 for use in skeletally mature adolescents and adults with giant cell tumor of bone (GCTB) if unresectable or when resection could result in severe morbidity [1, 2]. Osteoclastic giant cell-rich tumors of bone (GCRTB) are a distinct group of rare benign bone and cartilage tumors composed of aneurysmal bone cyst (ABC), giant cell tumor of bone (GCTB), and nonossifying fibroma ([3]; WHO 2020). In these bone pathologies and in the case of giant cell granuloma (GCG), denosumab is used as an off-label drug, if the case is inoperable, to reduce the volume of the lesion and consequently to reduce local compression, pain, functional deficits, and, when possible, to allow surgery of initially inoperable lesions. This kind of lesion is rare, with an incidence of 0.14–1.1 per million cases/year, occurring more frequently in the first 2 decades of life [2]. The aim of our study is to evaluate the efficacy and toxicity of denosumab in children diagnosed with GCRTB and treated in our center.

## 2. Patients and Methods

**Figure 1:**
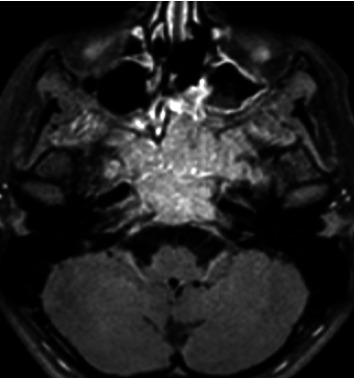
Before treatment, the T1 FATSAT-weighted images post contrast media axial plane shows a voluminous solid lesion which originates from the body of the sphenoid bone.

**Figure 2:**
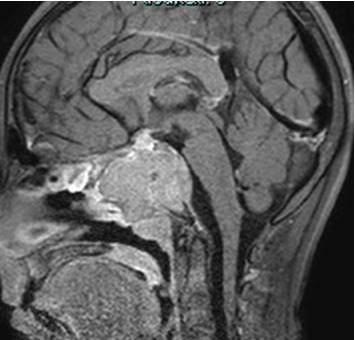
The voluminous solid lesion of the sphenoid bone before treatment in T1 FATSAT-weighted images postcontrast media sagittal plane.

From February 2022 to December 2023, five patients with GCRTB were treated with denosumab at the Istituto Giannina Gaslini. The age at diagnosis ranged from 8 to 17 years. Two of them were females and 3 males. Three patients had GCG [localized in the sphenoidal area (Figures 1 and 2), jaw, and maxilla, respectively], and two patients were affected by ABC (one localized in the right hemipelvis and one in D9 vertebra). Our local Ethics Committee approved denosumab as an off-label drug for use in all cases. Patients' parents signed the informed consent to treatment. Because of the possible side effects as hypocalcemia and jaw osteonecrosis, before starting therapy, each patient underwent blood sampling for an assay of calcium, vitamin D, and an evaluation of liver and renal function. Dental state was checked with an orthopantomography and a dental visit. Supportive oral therapy included calcium carbonate 500–1000 mg/die and vitamin D 1000 IU/die during the entire period of treatment. Denosumab was injected subcutaneously at a dose of 70 mg/m^2^ if the patient's weight was less than 50 kg or 120 mg/m2 if the patient's weight was ≥ 50 kg. Timing of injection was day +1, +8, +15, +28 and then one dose monthly for 3 months. A radiological assessment was scheduled at 1 month of therapy and then at 3–5 months according to clinical conditions, in order to decide whether to continue therapy after this time. Before every injection, a blood test was performed to assay calcium levels. Therapy was always administered in our day hospital, without routine premedication, and the patient stayed for about 45 min after the injection. All patients received 4 loading doses a week for 1 month. Overall, 21 doses of denosumab were administered to Patient 1, 20 doses to Patient 2, 11 doses to Patient 3, 10 doses to Patient 4, and 9 doses to Patient 5 (Table 1).

## 3. Results

**Figure 3:**
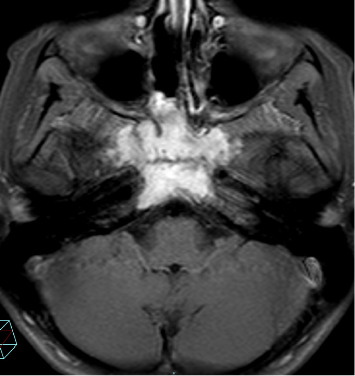
After treatment the T1 FATSAT-weighted images postcontrast media axial plane shows mass reduction in the body of the sphenoid bone.

**Figure 4:**
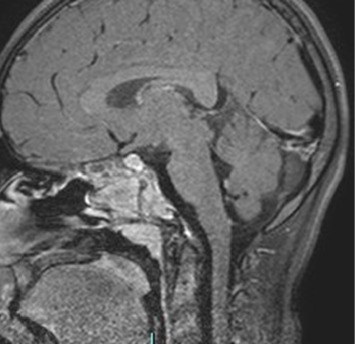
T1 FATSAT-weighted images postcontrast media sagittal plane after treatment shows a reduction in volume of the sphenoidal mass reducing compression of adjacent structures.

**Figure 5:**
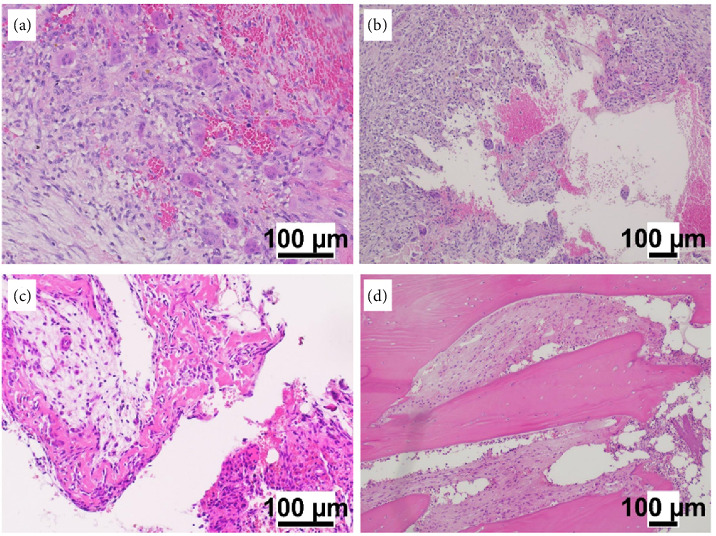
Microphotographs *(hematoxylin-eosin)*, (a, b) pretreatment, (c, d) post-treatment. (a) Rich cellular proliferation of both multinucleated giant cells and smaller round cells; (b) cavitated/cystic aspects of the lesion, with blood effusion; (c) depletion of the cellular component, with replacement by inflammatory/edematous tissue; (d) pauci-cellular fibrosis of the intraosseous component.

At the Giannina Gaslini Institute, in a period of 22 months, five patients under 18 years of age were diagnosed with GCRTB and received denosumab as an off-label drug in our day hospital. No acute or sub-acute adverse events were observed. All patients and their families showed good compliance with therapy. Radiological assessment was scheduled after the first 4 doses of denosumab, corresponding to 1 month of therapy, and then after 3–5 months, to evaluate whether there was an initial response in terms of reduction or stabilization of the disease, and to thus confirm the continuation of treatment. At +1 month, two patients (Patient 3 and Patient 5) underwent physical examination and not radiological examination due to family issues and objectively assessable clinical examination (maxilla and jaw). We observed volume reduction of lesions in 4 patients at different times of treatment (Table 2). Patient 1 (Figures 3 and 4) and Patient four showed a radiological response, while Patient 2 showed stabilization of disease, after the first 4 doses. Patient three and Patient five showed a radiological response at their first radiological evaluation, respectively, after 3 and 5 months from the start of therapy. At the last follow-up, Patients one, four, and five still showed a reduction of lesion volume, while patient two remained in stable disease. Unfortunately, Patient three was lost at the follow-up. No disease progression was observed. One patient (Patient 1) underwent surgery thanks to the significant reduction in the lesion after eight cycles of therapy. Histopathologically, alterations were observed in the lesion treated with denosumab, as shown in Figure 5, with depletion of the cellular component, replacement by inflammatory tissue, and pauci-cellular fibrosis of the intraosseous component, confirming the modifying action of denosumab, which clinically corresponds to the reduction or cessation of the growth of the lesion itself. To date, no patient showed side effects (Table 2). The maximum duration of therapy with denosumab was 22 months in Patient 1. This patient was the only one who received surgery, with successful results. For this reason, she subsequently received therapy every 6 weeks and not every 4 weeks like the other patients.

## 4. Discussion

The results obtained in our study showed that the use of denosumab can be helpful in some cases of inoperable GCRTB such as GCG and ABC [2]. Denosumab acts by blocking the RANKL signal pathway, which plays a role in the pathogenesis of some bone diseases [4]. The desirable effect of this action is the stabilization of the disease and the reduction of tumor size allowing previously unfeasible surgery due to lesion site and size. In our study, we obtained lesion reduction in four of five patients, and in one patient, surgery became possible without complications. These results confirm the possibility to reduce and stabilize the tumor with denosumab and the achievement of disease stabilization. As a neoadjuvant therapy, denosumab facilitates the en bloc resection of bone lesions thanks to size reduction, decrease of tumor vascularity, calcification, and formation of a sclerotic bone ring around the lesion [2, 5, 6]. However, neoadjuvant therapy is suggested for a maximum of 3–4 months in order to avoid excessive new bone formation and fibrosis, and thus to allow surgeons to perform an optimal curettage otherwise impossible if lesion margins become too hard [2, 7]. Denosumab can also be used as adjuvant therapy or to stabilize the disease. At present, denosumab injection is suggested every 4 weeks after the loading doses have been given during the first month every week; however, there is still no consensus on the timing of dose administration, duration of therapy, risk of recurrence at stop therapy, or side effects in children, as most reported data concern adult patients. Astrid Lipplaa et al. mentioned a trial in which prolonged use of denosumab was adopted until disease progression or documented unacceptable toxicity, with an interval dose administration of 12 weeks, after 1 year of therapy with monthly injection [2, 6, 8]. The optimal duration of treatment has not been defined yet. In the literature, the longest reported period of treatment was 5 years [9]. The risk of developing side effects related to prolonged treatment must be carefully considered and checked, such as osteonecrosis of the jaw, atypical femur fracture, severe hypocalcemia [2, 10], arthralgia, headache, nausea, fatigue, pain, anemia [11–13], and even hypercalcemia, that can occur from 7 weeks to 5 months after stopping the therapy [14]. Without a common consensus, the end of treatment is decided according to the patients' clinical conditions and the duration of administration. At stop therapy, a wait-and-see approach must be adopted, with radiological monitoring and strict follow-ups, because of the local aggression of these kinds of bone lesions, the high rate of local recurrence, and the possibility, though rare, of transformation into a malignant tumor [4, 9, 12]. Furthermore, even after surgery or the achievement of stable disease, the risk of recurrence remains and it is reported in literature in a very variable percentage according to treatment and site of disease [12]. Many recurrences arise within 7–9 months after stopping treatment [6]. Jiang C et al. reported that the median time to relapse after surgical intervention was 19.5 months (range 16.8–23.1), all patients were off therapy at the time of recurrence, and all of them received adjuvant denosumab after resection for a variable period that did not influence recurrence (from 6 to 12 months) [8]. Denosumab caused only minimal inhibitory effects on stromal cell lines and did not cause any apoptosis; this was hypothesized to explain why denosumab did not reduce the rate of recurrence [1, 6]. Literature data on this issue are discordant. Some authors reported a lower rate of recurrence with the use of denosumab [2, 15]. To date, all patients in our study are still receiving treatment and no relapses have been observed.

Other possible treatments have been reported. Over the past decades, sometimes, radiotherapy was performed as adjuvant therapy or to treat recurrence, but the local beneficial effect is debatable, with the risk of a secondary malignancy. For this reason, its use could be considered in elderly inoperable patients [9]. In our patients, radiotherapy was not proposed. As an alternative, the use of bisphosphonates was reported as systemic therapy in the case of inoperable lesion. Bisphosphonate zoledronic acid can stabilize disease by blocking the resorption capacity of reactive osteoclast-like giant cells, with a hypothesized apoptotic effect on neoplastic stroma [10, 12]. As reported by Lizz van H et al., a trial directly comparing denosumab and zoledronic acid included 39 retrospective patients affected by GCTB, 20 of them receiving the monoclonal antibody and 19 receiving zoledronic acid; no statistical difference was observed in radiological or clinical outcome [10]. After the first result with the introduction of denosumab in inoperable GCTB, clinical research on bisphosphonates became overshadowed, resulting in a lower number of publications [10].

## 5. Conclusion

The optimal treatment for the GCRTB is often a controversial issue. To date, surgery remains the treatment of choice. An important role of systemic therapy with denosumab as neoadjuvant therapy before surgery and for stabilization of lesions as long as possible was reported [12]. However, it seems that the use of denosumab does not prevent relapse and side effects have been reported in case of prolonged use, as major hypercalcemia and osteonecrosis in the jaw [12, 13]. The optimal treatment duration and interval between administrations are still debated and are presently decided on a case-by-case basis, according to recent literature reports. Our study confirms that denosumab is effective as neoadjuvant therapy or to stabilize lesions in children with inoperable GCRTB. Good compliance and tolerance were observed even in cases of prolonged therapy. No side effects were observed. Further analysis of side effects at the stop of therapy is to be analyzed, especially with regard to rebound osteoclastic activity. These results are preliminary and other studies are necessary in a larger number of cases and with a longer follow-up (3–5 years) period, even including data from other pediatric centers with the aim of producing common guidelines, since few data are available in the literature.

## Figures and Tables

**Table 1:** This table summarizes the characteristics of the 5 patients treated with denosumab years (y), patient (pt), giant cell granuloma (GCG), aneurysmal bone cysts (ABC).

Pt	Age at start of denosumab (y)	Sex	Disease	Involved bone	Dose (mg)	Total doses
pt 1	10	F	GCG	Sphenoid bone	90	21
pt 2	13	F	ABC	D9 vertebra	120	20
pt 3	17	M	GCG	Jaw	120	10
pt 4	9	M	ABC	Right hip	70	11
pt 5	14	M	GCG	Maxilla	100	9

**Table 2:** This table summarizes time-related treatment responses.

Pt	Radiological response + 1 month	Radiological response + 3/5 months	Radiological response at last follow-up (FU) in m^∗^	Surgery
pt 1	Reduction	Reduction	Yes (+22 m)	YES^***∗∗***^
pt 2	Stable disease	Stable disease	Stable disease (+15 m)	NO
pt 3	—	Reduction	Lost at FU (+6 m)	NO
pt 4	Reduction	Stable disease	Yes (+8 m)	NO
pt 5	—	Reduction	Yes (+5 m)	NO

*Note:* Patient (pt).

^∗^Months from start denosumab.

^∗∗^After 8 doses of denosumab.

## Data Availability

The data that support the findings of this study are available from the corresponding author upon reasonable request.
